# Intracranial response to positive end-expiratory pressure is influenced by lung recruitability and gas distribution during mechanical ventilation in acute brain injury patients: a proof-of-concept physiological study

**DOI:** 10.1186/s40635-025-00750-y

**Published:** 2025-04-14

**Authors:** Reka Bencze, Rafael Kawati, Anders Hånell, Anders Lewen, Per Enblad, Henrik Engquist, Kristin Jona Bjarnadottir, Odin Joensen, Annelie Barrueta Tenhunen, Filip Freden, Laurent Brochard, Gaetano Perchiazzi, Mariangela Pellegrini

**Affiliations:** 1 Anesthesia, Operation and Intensive Care Medicine, Uppsala University Hospital, Uppsala, Sweden; 2 Hedenstierna Laboratory, Department of Surgical Sciences, Uppsala University, Uppsala, Sweden; 3 Department of Medical Sciences, Section of Neurosurgery, Uppsala University, Uppsala, Sweden; 4 Keenan Centre for Biomedical Research, Critical Care Department, St. Michael’s Hospital, Unity Health Toronto, Toronto, Canada

**Keywords:** Acute brain injury, Mechanical ventilation, Lung–brain interaction, Intracranial pressure, Lung mechanics

## Abstract

**Background:**

The effect of positive end-expiratory pressure (PEEP) on intracranial pressure (ICP) dynamics in patients with acute brain injury (ABI) remains controversial. PEEP can benefit oxygenation by promoting alveolar recruitment, but its influence on ICP is complex. The primary aims of this study were to investigate 1) how lung recruitability influences oxygenation and 2) how lung recruitability and regional gas distribution, measured via recruitment-to-inflation (RI) ratio and electrical impedance tomography (EIT), affect ICP in response to PEEP changes in critically ill patients in their early phase of ABI.

**Methods:**

Ten mechanically ventilated ABI patients were included. Pressure reactivity index (PRx) was estimated. Using RI manoeuvre and EIT, lung recruitability and gas distribution were assessed in response to a standardised PEEP change (from high to low levels, with a delta of 10 cmH_2_O). Changes in ICP (ΔICP) were calculated between high and low PEEP. Lung inhomogeneity indices (global inhomogeneity index [GI] and local inhomogeneity index [LI]) were derived from EIT. Correlations between ventilatory variables and ICP were analysed.

**Results:**

Blood oxygenation significantly decreased, going from high (14 [IQR: 12–15] cmH₂O) to low (4 [IQR: 2–5] cmH₂O) PEEP. Reducing PEEP significantly increased ICP (from 9 [IQR: 5–13] to 12 [IQR: 8–16] mmHg, p < 0.01), while cerebral perfusion pressure (CPP) improved (from 71 [IQR:67–83] to 75 [IQR: 70–84] mmHg, p = 0.03) and mean arterial pressure (MAP) increased (from 79 [IQR: 69–95] to 84 [IQR: 76–99] mmHg, p < 0.01). The RI ratio correlated significantly with ΔICP (rho = 0.87, p < 0.01), as did Vrec% (proportion of recruited volume, rho = 0.65) and GI (rho = 0.5). LI did not correlate with ΔICP. PRx was 0.30 [IQR: 0.12–0.42], indicating a deranged cerebral autoregulation.

**Conclusions:**

Patients with a higher potential for lung recruitability had a more beneficial effect of PEEP on oxygenation. These effects should be interpreted cautiously, given that lung recruitability and global inhomogeneity of gas distribution significantly influenced the intracranial response to PEEP in ABI patients. As indicated by MAP and CPP, PEEP may impact systemic haemodynamics and cerebral perfusion when cerebral autoregulation is deranged. These findings underscore the importance of multimodal (i.e. respiratory, cerebral and haemodynamics) monitoring for optimising ventilation strategies in ABI patients and provide a framework for future research.

*Trial registration* Registration number: NCT05363085, Date of registration: May 2022

**Supplementary Information:**

The online version contains supplementary material available at 10.1186/s40635-025-00750-y.

## Background

A prompt admission to neurointensive care followed by neuroprotective interventions is critical to the successful management of acute brain injury (ABI) [1, 2]. Although invasive mechanical ventilation is a necessary part of the neuroprotective strategy and prevention of secondary hits [3, 4], it can harm the brain due to complex, unmonitored physiological interplay between intrathoracic and intracranial compartments [5]. The intracerebral effects of positive intrathoracic pressure in ABI patients have been much debated, and the evidence is still inconclusive [6]. High airway pressure, particularly the influence of positive end-expiratory pressure (PEEP), was initially assumed to elevate intracranial pressure (ICP) invariably. Intrathoracic pressure may affect venous cerebral outflow and, as such, negatively affect ICP [7].

Although not always fulfilling all acute respiratory distress syndrome (ARDS) criteria [8], mechanically ventilated ABI patients are prone to lung collapse caused by concomitant factors, such as deep sedation, neurogenic pulmonary oedema or aspiration pneumonia [9]. As such, optimisation of PEEP to avoid lung collapse is highly relevant. It has been recently shown that if the recruitment of alveolar units follows a rise in PEEP, ICP does not increase or even decrease, while gas exchange improves [10–12]. Following improved lung compliance and reduced shunt, a further reduction in ICP is expected when PEEP recruits the lung. The influence of regional lung overdistension and collapse on the propagation of alveolar pressures to the thoracic cavity and, consequently, to the brain has never been previously investigated.

To test lung recruitability, the recruitment-to-inflation (RI) manoeuvre has been recently introduced in clinical practice [13, 14]. The RI ratio represents the ratio between the recruited lung and the “baby lung” compliance. It differentiates the potential of lung recruitability from the inflation of the already open lung across a standardised 10 cm H_2_O change in PEEP. This simplified manoeuvre gives one global value of the RI ratio for the whole lung.

Electrical impedance tomography (EIT), a non-invasive bedside lung-imaging technique, has the potential for real-time monitoring of the regional distribution of lung ventilation and its changes following the modification of ventilatory settings [15]. It provides information about the inhomogeneity of gas distribution within the lungs. Therefore, EIT imaging combined with the RI ratio manoeuvre may provide information about the potential of regional lung recruitability and the inhomogeneity of gas distribution following changes in PEEP.

The primary aims of this study were to investigate how lung recruitability influences oxygenation and how lung recruitability and regional gas distribution affect ICP in response to PEEP changes in critically ill patients in their early phase of ABI.

## Methods

The study was approved by the Swedish National Ethical Review Authority (2020–07227) and conducted following the Helsinki Declaration and its subsequent revisions. It included mechanically ventilated patients within 72 h of invasive mechanical ventilation in an early phase of ABI. Patients were admitted to the neurosurgical intensive care unit (Neuro-ICU) of the Uppsala University Hospital, Sweden. As the patients were unconscious at the time of inclusion, informed consent was obtained from the patient’s next of kin. A detailed description of the methods is reported as supplementary material.

### Inclusion and exclusion criteria

Patients were prospectively screened for eligibility according to predefined inclusion criteria: 1) age older than 18; 2) ABI within 72 h from inclusion, i.e. subarachnoid haemorrhage; subdural haemorrhage; intracranial haemorrhage; traumatic brain injury; 3) less than 72 h since initiation of mechanical ventilation and insertion of invasive intracerebral pressure monitoring device; 3) mechanical ventilation expected to last for longer than 72 h; 4) ongoing ICP measurement. Exclusion criteria were: 1) chest tube or open chest trauma; 2) absolute contraindication to the insertion of nasogastric catheters: e.g., oesophagus rupture and oesophageal bleeding; 3) contraindication for the use of EIT: pacemaker and implantable cardioverter defibrillator, pregnancy, thoracic skin lesion, and burns on the belt area; 4) hemicraniectomy.

### Monitoring procedures

At inclusion, a nasogastric catheter for oesophagus pressure (Pes) monitoring (14Fr, Nutrivent, Sidam) was placed. At the moment of data acquisition, the oesophageal catheter and a patient spirometry catheter (D-lite + , GE Healthcare) with 9.5 ml instrumental dead space, not significantly interfering with patient CO_2_ clearance, were connected to the Pulmovista 500 pressure transducer (Pressure Pod, Dräger). Based on clinical routine, all patients had a central line catheter for drug infusion and central venous pressure (CVP) monitoring and an arterial catheter for invasive systemic blood pressure monitoring. Following clinical practice for intubated patients, side-stream capnography was recorded, and end-tidal carbon dioxide (EtCO_2_) was continuously monitored. All respiratory, haemodynamic, and neuromonitoring variables, including EIT imaging, were simultaneously acquired and synchronised offline for data analysis using MatLab, R2023b (The Mathworks, Natick, MA, USA). Demographics, epidemiologic, and clinical data were obtained from electronic medical records.

### Patient management during data collection

For data acquisition, all patients were placed in a semi-recumbent 30° head-elevated position and continuously sedated according to local standard practice. Deep sedation and complete neuromuscular blockade were established before data acquisition. The intraventricular drain catheter was closed throughout the protocol for ICP recording. An EIT belt was placed to collect chest impedance changes related to lung ventilation (Pulmovista 500, Dräger, Germany). The EIT belt was in place only during data acquisition and then removed, not interfering with clinical procedures. We strictly aligned with neuroprotection care bundle principles and always prioritised patient safety and best care. Data were not collected in case of severe respiratory, haemodynamic, or neurologic instability.

### Protocol and data collection

The ventilation mode was first changed to volume-controlled with a tidal volume of 6–8 ml/kg predicted body weight (PBW). The respiratory rate was adjusted to keep EtCO_2_ constant and maintain the clinical target of arterial pressure of carbon dioxide (PaCO_2_) unchanged. The fraction of inspiratory oxygen (F_I_O_2_) was set to stay partial pressure of arterial oxygen (PaO_2_) higher than 90 mm Hg. Data recording was initiated, and a standardised lung recruitment manoeuvre was performed, followed by a decreasing PEEP titration. Baseline PEEP was set based on best static compliance. After PEEP titration, an inspiratory-hold and an expiratory-hold manoeuvre were performed. A low-flow (5 L/min) inflation starting from PEEP 0 cm H_2_O and reaching the patient’s set tidal volume was performed to identify airway opening pressure (AOP), as previously described [16]. After baseline ventilation and reduction of respiratory rate to 10 breaths per minute to exclude the onset of intrinsic PEEP, patients were exposed to a single-breath derecruitment manoeuvre from high to low PEEP, with a delta of 10 cm H_2_O to calculate the RI ratio as previously described [13, 14]. Each PEEP step was set for at least three minutes, and plateau pressure was measured at low PEEP at the end of the manoeuvre. The RI ratio was spirometrically computed, and EIT images were acquired for a subsequent offline analysis of the recruited and inflated volumes. EIT images and other analysed variables were selected for each PEEP level during steady-state conditions.

### Analysis of respiratory variables

The end-expiratory transpulmonary pressure (Ppl) was directly calculated by subtracting Pes from PEEP [17]. This estimation of Ppl greatly reflects the dependent lung regions and is, as such, more representative of lung collapse [18]. Given its importance in promoting the transmission of static respiratory pressures to the intrathoracic and intracranial cavities [19, 20], chest wall elastance and its ratio to respiratory system elastance (E_CW_/E_RS_), as well as lung elastance (E_L_) where E_RS_ = E_CW_ + E_L_, were calculated. Given its clinical impact in mechanically ventilated patients with ABI [8], mechanical power was calculated as in Gattinoni et al. [21].

### Analysis of neuromonitoring variables

Cerebral perfusion pressure (CPP) was calculated by subtracting ICP from mean arterial pressure (MAP). The pressure reactivity index (PRx), an indicator for cerebral autoregulation, was calculated as previously described [2]: a moving Pearson correlation coefficient between MAP and ICP averaged over 10 s, using a 5-min moving time window. Thereafter, the values collected during a time interval of 1 h before the protocol started were averaged. A PRx value higher than 0.2 reflects poor cerebral autoregulation. The ICP pulse waveform analysis in the time domain was conducted to identify the second peak (P2), also called the tidal wave of the ICP curve, which is a proxy for intracranial compliance [22].

### Analysis of haemodynamics and other variables

**Fig. 1:**
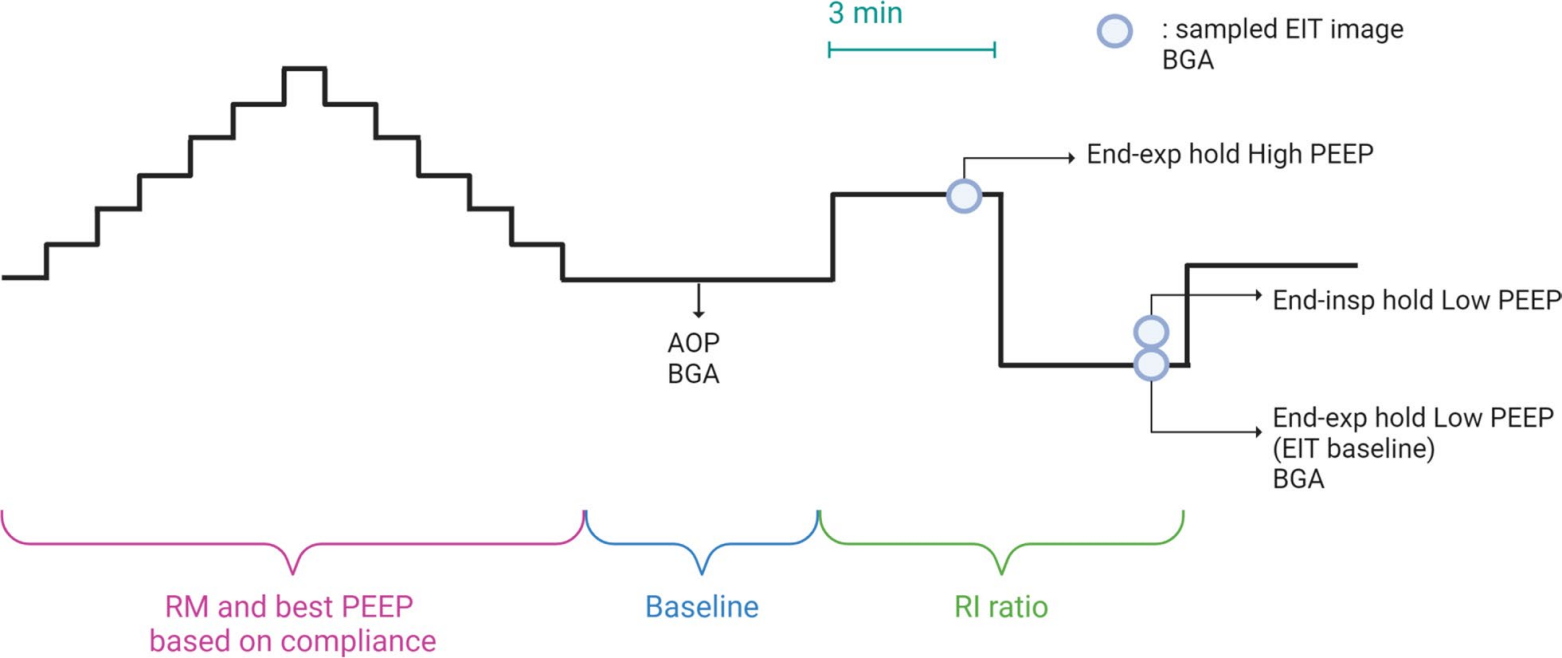
Study protocol for the primary analysis and estimation of EIT-based RI ratio. RI ratio with tested ΔPEEP equal to 10 cm H_2_O. *BGA* blood gas analysis, *EIT* electrical impedance tomography, *PEEP* positive end-expiratory pressure, *AOP* airway opening pressure, *RI* ratio recruitment–inflation ratio

MAP was calculated as the sum between 1/3(systolic blood pressure) and 2/3(diastolic blood pressure). Arterial and central venous blood samples were collected three times during the protocol: 1) at least 5 min after the lung recruitment manoeuvre, 2) at high PEEP, and 3) at low PEEP during RI manoeuvre. Based on blood gas analysis, the ventilatory ratio, estimating the dead space fraction, was calculated as: (minute ventilation [ml/min] × PaCO_2_ [mm Hg]) / (PBW × 100 [ml/min] × 37.5 [mm Hg]) [23]. The shunt fraction was estimated based on the venous admixture determination, considering the central venous oxygen saturation as an acceptable surrogate for mixed venous oxygen saturation and assuming the respiratory ratio equal to 0.8 [24]. Figure 1 provides a schematic representation of the protocol

### Analysis of EIT data

#### A. The RI ratio

**Fig. 2:**
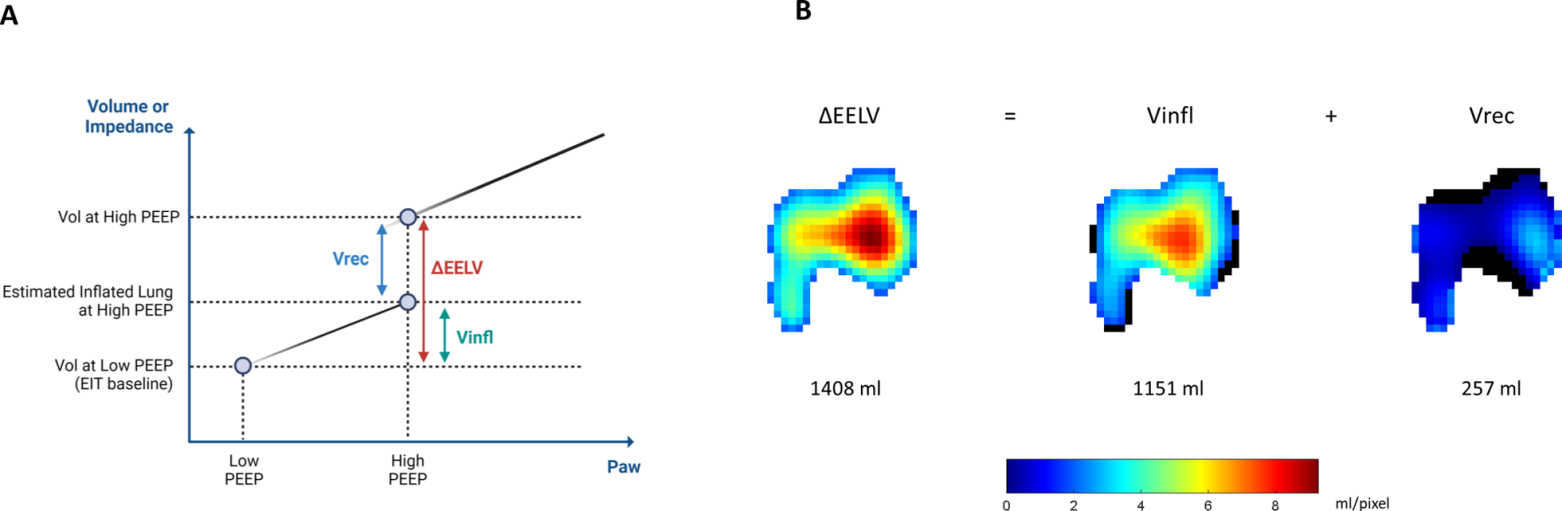
Method to calculate delta end-expiratory lung volume (ΔEELV), inflated volume (Vinfl) and recruited volume (Vrec). **A** Graphic representation of the three analysed volumes on the pressure–volume axes. **B** Representative example of spirometric volume and volume maps. The latter provides pixel-wise information about the analysed volumes. ΔEELV = Vinfl + Vrec, where ΔEELV = (EIT-image during expiratory-hold High PEEP) – (EIT-image during expiratory-hold LOW PEEP); Vinfl = [(EIT-image during inspiratory-hold Low PEEP)/ (plateau pressure – low PEEP)] x (high PEEP); Vrec = ΔEELV- Vinfl. Abbreviations: *ΔEELV* delta end-expiratory lung volume; *Vinfl* inflated volume; *Vrec* recruited volume, *EIT* electrical impedance tomography, *PEEP* positive end-expiratory pressure

The EIT baseline was set to correspond to the dynamic image with the lowest global impedance during expiratory hold at low PEEP. After that, dynamic EIT images (32 × 32 matrix) corresponding to 1) inspiratory hold PEEP low and 2) expiratory hold PEEP high were selected. For both selected EIT images, pixels characterised by delta impedance values (ΔZ) below 20% of the highest ΔZ in the same EIT image were considered not ventilated [25–27]. Based on the set tidal volume, impedance changes were translated into corresponding millilitres of gas to obtain the regional (pixel-based) distribution of ventilation [28]. Based on these EIT-derived images and the physiological rationale behind the RI ratio manoeuvre, the following three spirometric volume and volume maps, the latter providing pixel-wise information, were computed (see Figs. 1 and 2):

1. Delta end-expiratory lung volume (ΔEELV): the global change in lung volume corresponding to a delta PEEP of 10 cm H_2_O, between PEEP high and PEEP low and calculated as follows:

ΔEELV = (EIT image during expiratory hold PEEP high) – (EIT image during expiratory hold PEEP low)

 where (EIT-derived image during expiratory hold PEEP low) corresponds to the EIT baseline.

2) Inflated volume (Vinfl) : the portion of ΔEELV inflating the already opened lung when going from PEEP low to PEEP high and calculated as:

Vinfl = [(EIT image during inspiratory hold PEEP low—EIT image during expiratory hold PEEP low)/ (plateau pressure – PEEP low)] x (PEEP high – PEEP low).

where [(EIT image during inspiratory hold PEEP low—EIT image during expiratory hold PEEP low)/ (plateau pressure – PEEP low)] is the compliance of the lung open at PEEP low.

3) The recruited volume (Vrec): the portion of ΔEELV recruiting new portions of lung parenchyma when going from PEEP low to PEEP high and calculated as:

Vrec = ΔEELV- Vinfl. The Vrec was then expressed as a percentage (Vrec%) of the corresponding ΔEELV.

#### B. Regional compliance

**Fig. 3:**
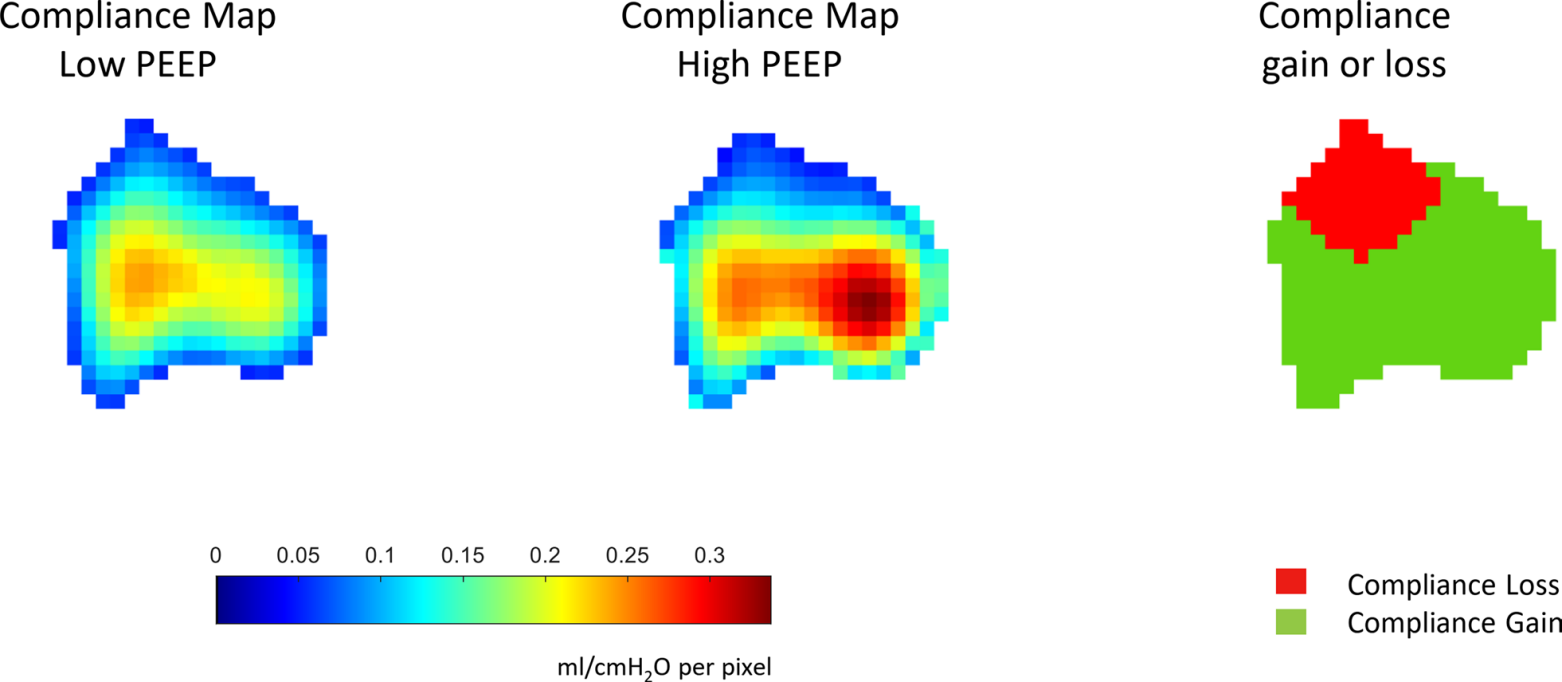
Compliance maps at high and low PEEP and compliance gain and loss based on EIT-analysis. Representative example. For compliance maps at low and high PEEP (left and central figures): the tidal change of ventilation for both PEEP is divided by the corresponding driving pressure. Pixel compliance is reported as ml/cmH_2_O per pixel. For compliance gain or loss (right figure). Gain is intended as an increase in compliance going from low to high PEEP. *Red* loss; *green* gain in compliance. *PEEP* positive end-expiratory pressure, *EIT* electrical impedance tomography

To further investigate regional lung recruitability and confirm lung recruitment and inflation at the two tested PEEP, regional maps of compliance at both low and high PEEPs have been computed based on the tidal impedance changes between plateau pressure and the corresponding PEEP [29]. The estimated tidal volume per pixel was then divided by the measured driving pressure to obtain the compliance maps (Fig. 3). Subsequently, the compliance map at high PEEP was subtracted from the compliance map at low PEEP to obtain the distribution of differential compliance. In this way, we discriminated pixels of compliance gain from pixels of compliance loss when lungs were exposed to high PEEP. The number of pixels with compliance gain was then expressed as a percentage of all pixels covering the lung area. Moreover, the mean compliance gain (or loss) was expressed in [ml/cm H_2_O per pixel]. The pixel compliance gain (or loss) summation was calculated.

#### C. EIT-based inhomogeneity index

To investigate the coupling between regional inhomogeneity of lung ventilation and neuromonitoring variables, two EIT-based indices were calculated for the ΔEELV delta volume maps: the global inhomogeneity index (GI) and the local inhomogeneity index (LI) [30]. These two indices report different information about gas distribution. GI is an index of global gas inhomogeneity [31, 32], while LI quantifies local inhomogeneity among neighbouring pixels. GI shows the variation of tidal volume distribution in the whole lung. GI is calculated as the summation of the absolute difference between the median value of the estimated gas volume for each analysed map and the estimated gas volume for every pixel. LI calculates the local differences among neighbour pixels. GI and LI were normalised by dividing them by the total gas content in the analysed volume map to make both indices comparable among patients. We hypothesised that non-uniformity of gas distribution may lead to alterations in cerebral venous outflow, impacting ICP.

GI and LI can be summarised with the following equations as in Zhao et al. [30]:

$$GI = \mathop \sum \limits_{x,y \in lung} \left| {Dgas_{xy} - Median \left( {Dgas_{lung} } \right)} \right|{ } \div \mathop \sum \limits_{x,y \in lung} Dgas_{xy} ,$$

$$LI = \mathop \sum \limits_{x,y \in lung} \left( { \frac{1}{m - 1} \times \mathop \sum \limits_{i,j \in - 1,1 \cap x + 1, y + i \in lung} \left| {Dgas_{xy} - Dgas_{x + i, y + j} } \right|} \right){ } \div \mathop \sum \limits_{x,y \in lung} Dgas_{xy} ,$$

where *x* and *y* are the coordinates of each pixel; Dgas_xy_ is the value of the differential impedance for the single pixel; Median (Dgas_lung_)is the median value of impedance in the lung area; m-1 is the number of neighbour pixels equal to eight; Dgas_x+i,y+j_ is the differential impedance for the neighbouring pixels of Dgas_xy_.

### Statistical methods

An a priori sample size calculation was not feasible as no studies investigated the association between EIT-derived indices of lung recruitability and intracranial pressure in brain-injured patients. Our sample size was comparable to previous physiologic studies regarding PEEP changes in ABI patients [11]. We investigated the association between ventilatory variables (i.e. spirometric RI ratio, percentage of recruitable lung and indices of regional inhomogeneity) and changes in ICP (ΔICP) going from low PEEP to high PEEP levels, where positive changes indicating a higher ICP at low PEEP compared to high PEEP.

The primary outcomes were to test:

- 1) The association between RI ratio and oxygenation (i.e. PaO_2_/F_I_O_2_).
- 2) The association between recruited volume and ΔICP.
- 3) The association between inhomogeneity of gas distribution (GI, LI) and ΔICP.

Secondarily, we investigated changes in respiratory (e.g., static compliance, and end-exp Ppl), neuromonitoring (i.e. ICP, CPP and P2), and haemodynamic (i.e. CVP and MAP) variables when exposed to two different PEEP levels.

Data were expressed as median and interquartile range (interquartile range, IQR) or mean (± standard deviation, SD). The correlation between different variables was assessed using Spearman’s rank correlation coefficient with a 95% confidence interval. F-test statistic (α = 0.05) was used for linear regression analysis. Wilcoxon matched-pairs signed-rank test was used to test statistically significant changes in respiratory and neuromonitoring variables between the two tested PEEP levels. In case of missing values in the data range, the whole pair was excluded from the analysis. Friedman's test, followed by Bonferroni’s correction, was used to test statistical differences for neuromonitoring parameters among baseline ventilation, high PEEP and low PEEP. Statistical analysis was performed using MatLab (MATLAB R2023b, MathWorks, MA, USA) and GraphPad (GraphPad Prism v10, California, USA).

## Results

### General findings

During the study period, 11 patients were assessed for inclusion. One patient was later excluded for unsuccessful placement of the oesophagal catheter and a newly diagnosed hiatus hernia. Ten patients (three females) with a median age of 62 [IQR:61–68] were included in the study; Table 1. Thirty per cent of patients had neurological diseases as previous comorbidities: two had a prior ischaemic stroke, and one had a subarachnoid haemorrhage. Among the most represented comorbidities, 60% of patients had hypertension, and 20% had cardiovascular diseases but no heart failure. Other demographics collected at patients’ inclusion are reported in Table 1. After the lung recruitment manoeuvre, the applied PEEP, selected based on the best static compliance, was equal to 8 [IQR:8–10] cm H_2_O, and no intrinsic PEEP was detected. Thirty per cent of patients needed a low-dose vasopressor infusion during data acquisition. Table 2 reports lung function after lung recruitment, indices of neuromonitoring and haemodynamic variables during baseline ventilation. Based on the baseline PaO_2_/FIO_2_ ratio (Table 2, and Table E1), three (30%) patients were potentially classified as mild or moderate ARDS.

**Table 1:** Characteristics of the patients at study inclusion

Variable		
Demographics		
Age [years], median [IQR]	62	[61–68]
Gender, female, n [%]	3	33%
BMI [kg/m^2^], median [IQR]	30	[24–30]
Comorbidities		
Respiratory disease, n [%]	1	10%
Cardiovascular disease, n [%]	2	20%
Neurological disease, n [%]	3	30%
Thrombotic events, n [%]	1	10%
Hypertension, n [%]	6	60%
Malignant disease, n [%]	1	10%
Diabetes mellitus, n [%]	1	10%
Medication		
Steroids, n [%]	1	10%
ACEi/ARB, n [%]	3	30%
Anticoagulant, n [%]	4	40%
Statins, n [%]	3	30%
Reason for NICU admission		
SAH, n [%]	7	70%
TBI, n [%]	1	10%
ICH, n [%]	2	20%
Glasgow Coma Scale	9	[7–11]
Type of ICP monitoring		
EVD, n [%]	8	80%
Parenchymal, n [%]	2	20%
Timing		
Time injury to ICU admission [hours]	7	[6–9]
Time ICU admission to inclusion [hours]	43	[37—52]
Time mechanical ventilation to inclusion [hours]	46	[42—53]

IQR: interquartile range; BMI: body mass index; ACEi: angiotensin-converting enzyme inhibitors; ARB: angiotensin receptor blockers; Neuro-ICU: neuro intensive care unit; SAH: subarachnoid haemorrhage; TBI: traumatic brain injury; ICH: intracranial haemorrhage; GCS: Glasgow Coma Scale; EVD: external ventricular drain

**Table 2:** Respiratory mechanics, neuromonitoring, and haemodynamics variables were measured during baseline ventilation

Variable		
	Median	IQR
Respiratory mechanics		
RI ratio	0.35	[0.14–0.43]
Tidal volume [ml] *(unchanged)*	420	[379–476]
Respiratory rate [bpm]	15	[15–17]
ePEEP [cm H_2_O]	8	[8–10]
totPEEP [cm H_2_O]	8	[8–10]
Minute ventilation [l/min]	7	[6–8]
End-exp Ppt [cm H_2_O]	1	[−4–4]
Static driving pressure [cm H_2_O]	7	[6–8]
Static compliance [ml/cm H_2_O]	70	[45 -80]
Chest wall elastance [cm H_2_O/l]	4.58	[4.35–4.96]
Mechanical power [J/s]	6	[5–8]
Neuromonitoring		
PRx	0.30	[0.12–0.42]
CPP [mm Hg]	79	[70–84]
ICP [mm Hg]	7	[4–10]
P2, ICP [mm Hg]	12	[10–14]
Haemodynamics and other variables		
Vasopressors need during data acquisition, n [%]	3	30%
Vasopressors during data acquisition when used [mcg/kg/min]	≤ 0.04	
MAP [mm Hg]	85	[81–93]
CVP [mm Hg]	9	[5–11]
EtCO_2_ [mm Hg]	37	[32–39]
PaCO_2_ [mm Hg]	40	[37–44]
Ventilatory ratio	1.1	[0.9–1.2]
Shunt fraction	0.1	[0.06–0.12]
pH	7.42	[7.38–7.44]
FIO_2_	0.3	[0.30–0.46]
PaO_2_ [mm Hg]	107	[100–125]
PaO_2_/FIO_2_ [mm Hg]	341	[229–430]
SaO_2_ [%]	98	[98–99]

Baseline ventilation was after the lung recruitment manoeuvre. Data were reported as median [interquartile range]. *RI ratio* recruitment inflation ratio, *ePEEP* external positive end-expiratory pressure, *totPEEP* total positive end-expiratory pressure, *PL* transpulmonary pressure, *PRx* pressure reactivity index, *CPP* cerebral perfusion pressure, *ICP* intracranial pressure, *P2*, *ICP* the second peak of intracranial pressure, *MAP* mean arterial pressure, *CVP* central venous pressure, *EtCO*_*2*_ end-tidal carbon dioxide, *PaCO*_*2*_ arterial partial pressure of carbon dioxide, *PaO*_*2*_ arterial partial pressure of oxygen, *SaO*_*2*_ arterial saturation of oxygen

There were no missing data for all respiratory and neuromonitoring variables but for EtCO_2_ (four patients out of ten) and arterial blood gas analysis-derived variables at low PEEP (three out of ten). The two PEEP levels applied to perform the RI manoeuvre were 14 [IQR:12–15] cm H_2_O and 4 [IQR:2–5] cm H_2_O, maintaining a consistent delta PEEP of 10 cm H₂O, following the RI manoeuvre protocol (Table 3).

**Table 3:** Respiratory mechanics, neuromonitoring, and haemodynamics variables were measured at high and low PEEP

Variable	PEEP high			PEEP low		p	
	Median		IQR	Median	IQR		
Respiratory mechanics							
ePEEP [cm H_2_O]	14		[12–15]	4	[2–5]	< 0.01	*
totPEEP [cm H_2_O]	14		[12–15]	4	[2–5]	< 0.01	*
Minute ventilation [L/min]	7		[6–8]	7	[6–8]	0.29	
End-exp Ppl [cm H_2_O]	2		[−1–4]	−5	[−8–−2]	< 0.01	*
Static driving pressure [cm H_2_O]	7		[6–8]	6	[6, 7]	0.42	
Static compliance [ml/cm H_2_O]	69		[57–82]	65	[50–78]	0.03	*
Chest wall elastance [cm H_2_O/l]	5.63		[5.39–8.13]	4.39	[4.10–6.42]	0.25	
Lung elastance [cm H_2_O/l]	8.63		[7.92–11.01]	12.13	[9.63–12.73]	0.2	
Chest wall elastance/respiratory system elastance (E_CW_/E_RS_)	0.37		[0.35–0.46]	0.30	[0.23–0.37]	0.05	*
Neuromonitoring							
CPP [mm Hg]	71		[67–83]	75	[70–84]	< 0.01	*
ICP [mm Hg]	9		[5–13]	12	[8–16]	< 0.01	*
P2 [mm Hg]	12		[10–15]	15	[12–21]	< 0.01	*
Haemodynamics and other variables							
MAP [mm Hg]	79		[69–95]	84	[76—99]	< 0.01	*
CVP [mm Hg]	10		[8–12]	8	[7–12]	0.02	*
EtCO_2_ [mm Hg]	42		[36–42]	42	[39–48]	0.62	
PaCO_2_ [mm Hg]	40		[37–44]	39	[37–46]	0.81	
Ventilatory ratio	1.04		[0.92–1.16]	1.09	[0.89–1.25]	1	
Shunt fraction	0.11		[0.09–0.22]	0.16	[0.12–0.25]	0.47	
pH	7.41		[7.38–7.44]	7.43	[7.37–7.44]	0.53	
PaO_2_ [mm Hg]	108		[100–121]	94	[90–100]	0.04	*
SaO_2_ [%]	98		[98–99]	97	[97–98]	0.07	

External high and low PEEP values correspond to the two PEEP levels selected during the single-breath derecruitment manoeuvre to calculate the RI ratio. Elastance-derived end-insp PL [cm H_2_O] calculated as (Pplat × E_L_/E_RS_) and Elastance-derived end-exp PL [cm H_2_O] calculated as (PEEP tot × E_L_/E_RS_). Data reported as median [IQR]. The Wilcoxon signed-rank test (α = 0.05) was used to detect statistical differences. *PEEP* positive end-expiratory pressure, *PL* transpulmonary pressure, *CPP* cerebral perfusion pressure, *ICP* intracranial pressure; *P2* second peak of intracranial pressure, *MAP* mean arterial pressure, *CVP* central venous pressure, *EtCO*_*2*_ end-tidal carbon dioxide, *PaCO*_*2*_ arterial partial pressure of carbon dioxide, *PaO2* arterial partial pressure of oxygen, *SaO*_*2*_ arterial saturation of oxygen, *Paw* airway pressure, *Pplat* plateau pressure, *E*_*L*_ elastance of the lung, *E*_*RS*_ elastance of the respiratory system

### Oxygenation variables

**Fig. 4:**
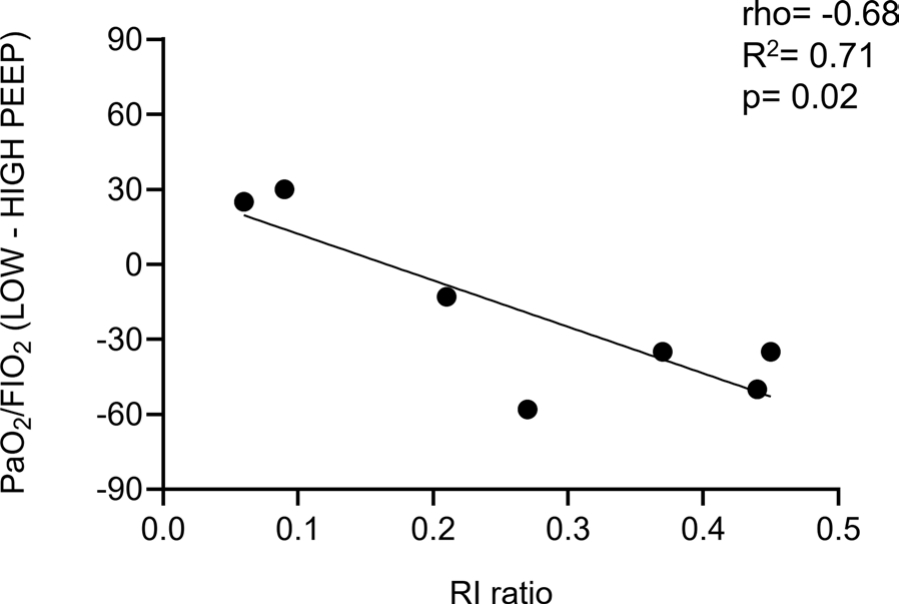
Linear regression and Spearman’s correlation investigating the relationship between the changes in PaO_2_/FIO_2_ and RI ratio. Negative ΔPaO_2_/FIO_2_ values indicate a reduction of PaO_2_/FIO_2_ going from high to low PEEP. The higher the recruitability (RI ratio), the more PaO_2_/FIO_2_ is reduced, going from high to low PEEP. Three out of ten patients had missing values for PaO_2_ at low PEEP. *FIO*_*2*_ fraction of inspiratory oxygen; *PaO*_*2*_ arterial partial pressure of oxygen, *RI* recruitment-to-inflation ratio; rho = Spearman correlation coefficient; R^2^: coefficient of determination; p = p-value or probability value

The FIO_2_ was 0.3 [IQR: 0.30–0.46] and kept constant throughout the study. The PaO_2_, but not the arterial saturation of oxygen (SaO_2_), significantly decreased from 108 [IQR: 100–121] mm Hg to 94 [IQR: 90–100] mm Hg when going from high to low PEEP. The changes in PaO_2_/FIO_2_ ratio when going from high to low PEEP were associated with the spirometric RI ratio: the higher the RI, the more the PaO_2_/FIO_2_ decreased at low PEEP (Fig. 4).

### Respiratory variables

**Fig. 5:**
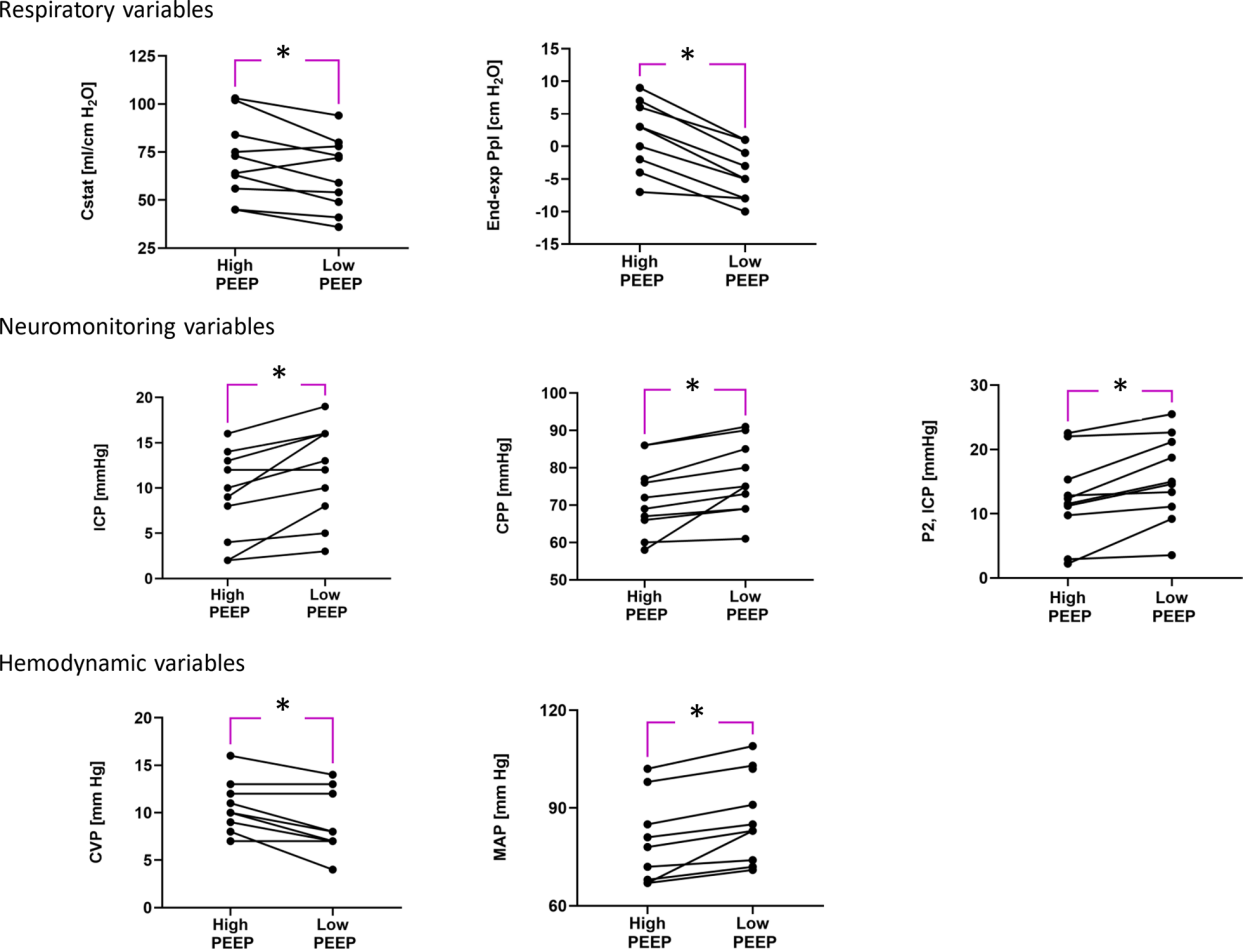
Individual changes of respiratory variables, neuromonitoring variables and haemodynamic variables, when changing PEEP. Following the RI manoeuvre, the PEEP changes from high to low. The reported respiratory variables are static compliance (Cstat), end-expiratory transpulmonary pressure (Ppl). The reported neuromonitoring variables are intracranial pressure (ICP), cerebral perfusion pressure (CPP) and the second peak of ICP as a proxy for intracranial compliance (P2). The reported haemodynamic variables are central venous pressure (CVP) and mean arterial pressure (MAP). *: indicate significant differences

The minute ventilation and the end-tidal carbon dioxide did not change significantly between high and low PEEP (p = 0.29). Static compliance significantly decreased when going from high to low PEEP. This was true for eight out of ten patients (Fig. 5). The end-expiratory Ppl significantly dropped from 2 [IQR: −1 to 4] cm H_2_O to −5 [IQR: −8 to −2] cm H_2_O when decreasing PEEP. The E_CW_/E_RS_ was equal to 0.37 [IQR: 0.35–0.46] at high PEEP (Table 3).

### Neuromonitoring variables

**Fig. 6:**
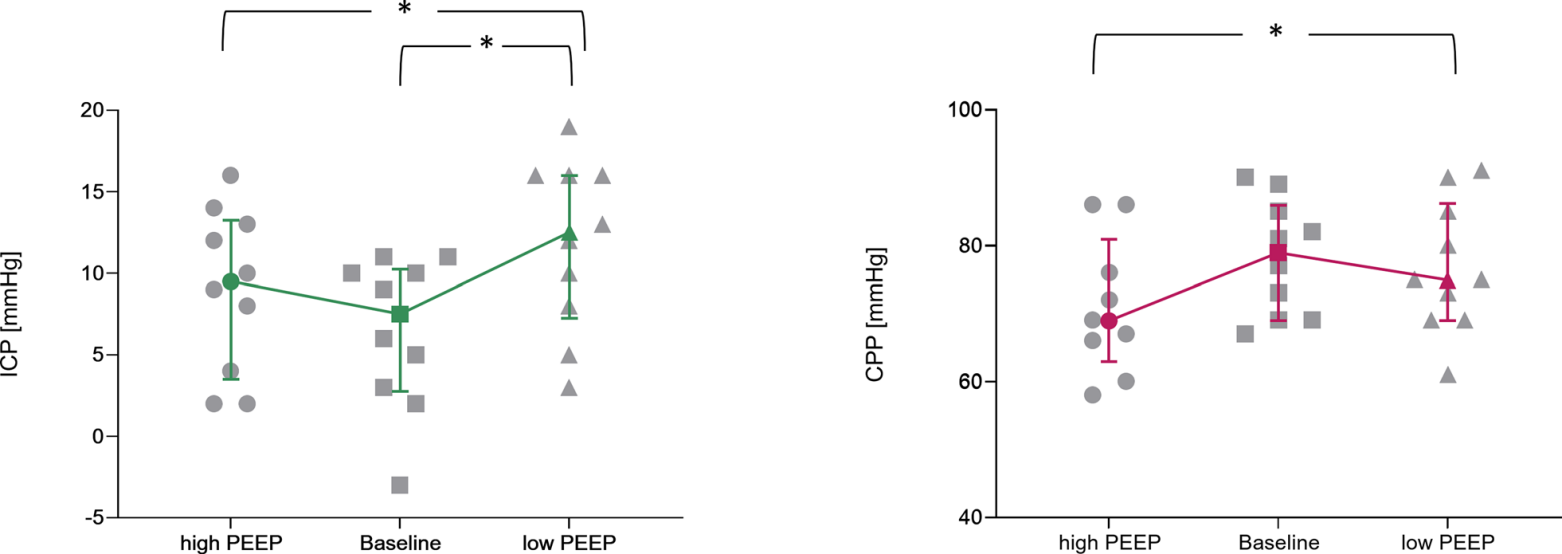
ICP and CPP at 1) PEEP high, 2) baseline ventilation, and 3) PEEP low. Data were reported as individual values (grey) and median with interquartile range (green). Abbreviations: *ICP* intracranial pressure, *CPP* cerebral perfusion pressure. Friedman’s test followed by Bonferroni *: to mark significant differences

CPP, ICP, and P2 increased significantly when PEEP was dropped from a high to a low level (Fig. 4). Compared to baseline ventilation, ICP did not change significantly when PEEP was set higher (7 [IQR:4–10] mm Hg vs 9 [IQR:5–13] mm Hg, p < 0.5); whereas, ICP was significantly higher at low PEEP if compared to ICP during baseline ventilation (7 [IQR:4–10] mm Hg vs 12 [IQR:8–16] mm Hg, p < 0.01) (Fig. 4 and Table E2). Despite CPP being significantly lower at high PEEP compared to low PEEP (71 [IQR:67–83] vs 75 [IQR:70–84] mm Hg, p = 0.03), it did not change significantly when comparing both CPP at high and low PEEP to CPP at baseline PEEP (79 [70–84] mm Hg) (Figs. 5 and 6, and Table E2). PRx was higher than 0.2 for 7 out of ten patients and its median value was 0.30 [IQR: 0.12–0.42]; Table 2.

### Haemodynamics and other variables

MAP was significantly higher at low PEEP compared to high PEEP, and this difference was independent of vasopressor use (Fig. 5 and Table 3). The ventilatory ratio, a surrogate of dead space, was kept in its normal range and remained unchanged at the two set PEEP levels. Although the change did not reach statistical significance, the estimated shunt fraction slightly increased when PEEP was lowered.

### Analysis of EIT data

#### A. The RI ratio

**Fig. 7:**
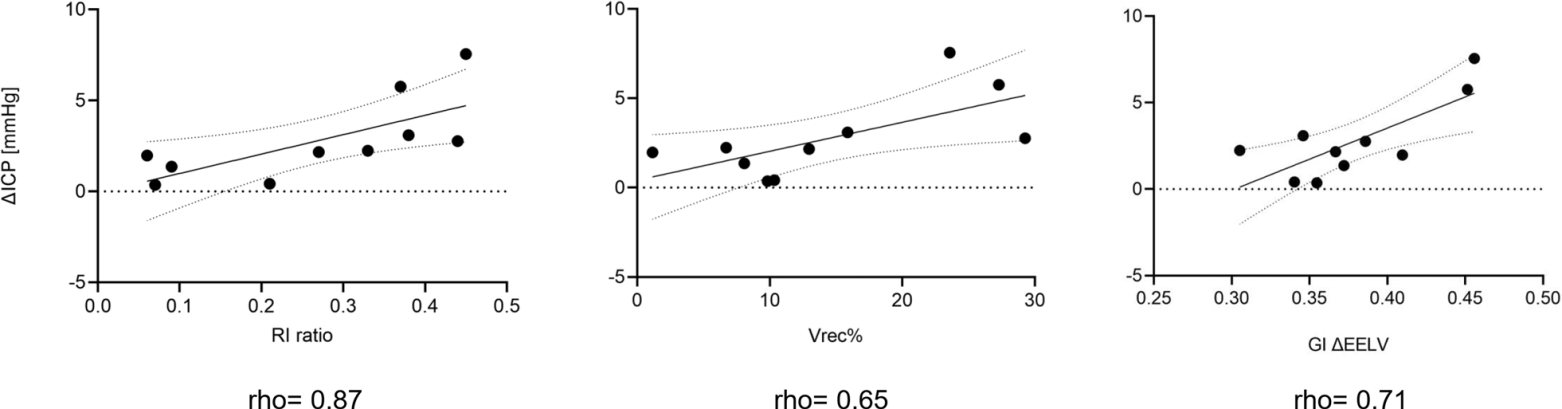
Linear regression and Spearman’s correlation investigating the relationship between ΔICP and 1) RI ratio, 2) Vrec%, and 3) GI of the ΔEELV image. Abbreviations: *ΔICP* changes in intracranial pressure going from the high to low PEEP level; *RI ratio* recruitment-to-inflation ratio; *Vrec%* recruited volume expressed as percentage of the total volume change (ΔEELV); *GI* global inhomogeneity calculated based on the EIT-derived the total volume change (ΔEELV) image; rho = Spearman correlation coefficient; R^2^: coefficient of determination; p = p-value or probability value

For all included patients, the AOP was equal to 0 cm H_2_O. The median value for the spirometric RI ratio was 0.35 [IQR: 0.14–0.43] (see Table 2). Following the derecruitment manoeuvre from high to low PEEP, the spirometric ΔEELV was estimated equal to 991 [SD: ± 320] ml, composed by an inflated volume, Vinfl equal to 841 [SD: ± 271] ml and a recruited volume, Vrec of 149 [SD: ± 110] ml (Figure E1, Table E3). Figure 2 reports representative examples of ΔEELV, Vinfl, and Vrec estimated based on EIT images. The spirometric RI ratio showed a significant association with ΔICP: both the tested linear regression (R^2^ = 0.51, p = 0.02) and Spearman’s correlation (correlation coefficient, rho = 0.87, p < 0.01); Fig. 7.

A significant association was demonstrated between Vrec% and the changes in ICP. The linear regression between Vrec% and ΔICP showed an R^2^ of 0.45 (p = 0.03); see Fig. 7.

### B. Regional compliance

The total number of pixels gaining compliance (58% of total pixels) was not different from the number of pixels losing compliance (42% of total pixels) while recruiting, changing from low to high PEEP (Table 4). On the other hand, the magnitude of compliance gain (recruitment) at high PEEP was significantly higher than the magnitude of compliance loss (overdistension) (13 [IQR: 9–21] ml/cm H_2_O of total compliance gain vs 6 [IQR:3–8] ml/cm H_2_O of total compliance loss, p = 0.02). Both linear regression and correlation analysis showed a significant coupling between the magnitude of compliance gain and the indices of lung recruitment investigated in the study (RI ratio and Vrec%) (Figure E2).

**Table 4:** EIT-based analysis on regional compliance

Variable			Variable			p	
	Median	IQR		Median	IQR		
Compliance gain [%]	58	[53–63]	Compliance loss [%]	42	[37–47]	0.11	
Compliance gain [ml/cm H_2_O per pixel]	0.07	[0.04–0.08]	Compliance loss [ml/cm H_2_O per pixel]	0.03	[0.02—0.05]	0.02	*
Sum of compliance gain [ml/cm H_2_O]	13	[9–21]	Sum of compliance loss [ml/cm H_2_O]	6	[3–8]	0.02	*

This analysis differentiates compliance gain (recruitment) from compliance loss (overdistension) at high PEEP compared to low PEEP. Data reported as median [interquartile range]. Compliance gain (or loss) expressed as [%] indicates the number of pixels with Compliance Gain (or Loss). Compliance gain (or loss) expressed as [ml/cm H_2_O per pixel] indicates the mean magnitude of compliance change per pixel. The sum of compliance gain (or loss) expressed as [ml/cm H_2_O] reports the summation of compliance gain (or loss) for all ventilated pixels. The Wilcoxon signed-rank test (α = 0.05) was used to detect statistical differences. *PEEP* positive end-expiratory pressure

### C. EIT-based inhomogeneity index

GI and LI were equal to 0.36 [SD: ± 0.03] and 0.11 [SD: ± 0.01], respectively.

A significant association was demonstrated between the GI and the changes in ICP; see Fig. 7, graph on the right. The linear regression between GI and ΔICP showed an R^2^ of 0.58 (p = 0.01). Unlike the GI index, the LI did not show a significant association with ΔICP. The linear regression between LI and ΔICP showed an R^2^ of 0.09 (p = 0.39).

## Discussion

The effect of PEEP on ICP dynamics in ABI patients remains debated [6]. While high PEEP is thought to increase ICP due to elevated intrathoracic pressure impairing cerebral venous outflow, this is not always the case. PEEP may be safe for ABI patients when promoting alveolar recruitment and oxygenation [11, 12]. Our findings suggest that other mechanisms contribute to the PEEP–ICP relationship, with a predominant influence on cerebral arterial inflow rather than venous outflow. Our findings can be summarised as follows:

1. Lung recruitability improves oxygenation and influences ICP dynamics.
2. PEEP can affect ICP primarily through cerebral arterial inflow.
3. Heterogeneous lung mechanics modulate the impact of PEEP on ICP.

1. Lung recruitability improves oxygenation and influences ICP dynamics.

The regulation of cerebral blood flow relies on overlapping and redundant mechanisms to ensure efficient oxygenation of individual cells [33]. Hypoxemia is, therefore, recognised as one of the primary preventable factors potentially contributing to secondary brain injury [4]. As expected [11, 12], increasing PEEP led to lung recruitment and improved oxygenation in our cohort. This was reflected by a higher ΔPaO₂ between high and low PEEP, particularly in patients with more recruitable lungs. However, SaO₂ remained stable, likely due to its low sensitivity to changes in oxygenation within the normal range.

Despite the low RI ratio (0.35 [IQR: 0.14–0.43]), our results suggested that a lung recruitability (Vrec%) of 15 ± 10% was enough to impact oxygenation and ICP in ABI patients. In our study, decreasing PEEP led to derecruitment, as confirmed by lower compliance and negative end-expiratory transpulmonary pressure. This lung collapse was paralleled by increased heterogeneity in gas distribution, which we found to be directly linked to changes in ICP. Specifically, greater recruitability and inhomogeneous gas distribution were associated with a more pronounced change in ICP (larger ΔICP) following a PEEP drop.

Interestingly, ICP and CPP were better preserved at baseline PEEP (set by optimal compliance) than during the recruitment manoeuvre, highlighting the complex interplay between ventilator settings and cerebral haemodynamics.

2) PEEP can affect ICP primarily through cerebral arterial inflow

The non-expandable cranial cavity comprises brain tissue, cerebral blood, and cerebrospinal fluid [7]. As such, in stable conditions, the balance between cerebral arterial inflow and cerebral venous pressure governs the dynamics of ICP and cerebral blood flow. Positive-pressure mechanical ventilation, mainly static changes in ventilatory pressure (i.e. PEEP), along with pulmonary and chest wall elastance, plays a central role in transmitting intrathoracic pressures to the intracranial compartment, influencing cerebral venous outflow and arterial inflow. Our results suggested that PEEP-induced ICP changes were primarily driven by effects on cerebral arterial inflow rather than venous congestion. MAP and CPP decreased at high PEEP, while intracranial compliance (reflected by a lower P2 waveform component) increased. This indicates that higher PEEP reduced cerebral blood flow in patients with impaired autoregulation, leading to a secondary reduction in ICP. Importantly, this should not be interpreted as improved cerebral perfusion but rather as a consequence of reduced systemic pressure and cerebral blood flow.

The transmission of intrathoracic pressures to the brain depends on pulmonary and chest wall elastance [19, 20]. In our cohort, the high E_CW_/E_RS_ ratio at high PEEP suggested an increased chest wall stiffness, leading to elevated pleural pressure and reduced left heart afterload. This likely contributed to the observed drop in arterial pressure and CPP. Conversely, the non-significant change in E_L_ observed going from high to low PEEP suggested the absence of a meaningful influence on right heart afterload [34].

*PEEP and cerebral venous outflow*. The impact of intrathoracic pressure on cerebral outflow is the most well-documented mechanism through which positive-pressure mechanical ventilation affects ICP [35]. A safety threshold is set by a vascular waterfall mechanism (or Starling resistor) created by collapsible cerebral veins following the patent sagittal sinus exiting the rigid skull [36]. In neuro-ICU patients, the raised ICP and the head-elevated position facilitate the closure of the waterfall, impeding the transmission of the intrathoracic pressure and CVP to the brain and protecting the latter from further pressure increases [37]. Suppose the intrathoracic pressure exceeds this safety threshold (ICP-CVP, set at 2.5 mm Hg in ventilated patients with 30-degree head elevation); the collapsible cerebral veins will stay patent and allow retrograde pressure transmission to the brain. Moreover, previous studies report that a PEEP increase, up to 15–20 cm H_2_O, does not necessarily impact, or can even reduce, ICP if cerebral compliance is not compromised [5, 38, 39]. It has also been demonstrated that a CVP exceeding 20 cm H₂O may be required to completely reopen the jugular veins [40]. In our study, the CVP overcame the ICP at high PEEP, suggesting a possible negative impact on cerebral venous outflow. An expected consequence of this increase in CVP would be a further elevation of ICP. Conversely, in our study, high PEEP led to a significant decrease in ICP compared to low PEEP, likely due to a predominantly negative effect on cerebral arterial inflow.

*PEEP and cerebral arterial inflow*. The influence of PEEP on cerebral arterial inflow, explaining the reduction in ICP observed in our patient's cohort, is indirectly suggested by changes in systemic arterial pressure, CPP, and P2. In our cohort, the increase in PEEP transmitted to the thoracic cavity (i.e. causing an increase in pleura pressure) negatively influences right heart end-diastolic volume and left heart afterload, reducing cardiac output [33]. The significant reduction in systemic arterial pressure, and consequently of CPP, observed in our study indirectly reflected a negative influence of PEEP on systemic haemodynamics, which, given the impaired autoregulation, results in a reduction of cerebral blood flow. To the best of our knowledge, no studies, except for one currently ongoing [41], have examined the impact of mechanical ventilation on P2 and intracranial compliance [42]. An impaired cerebral autoregulation, as observed in our study, resulting in insufficient cerebral vasoconstriction despite a reduction in cardiac output, may account for the increased cerebral compliance observed at high PEEP compared to low PEEP.

3) Heterogeneous lung mechanics modulate the impact of PEEP on ICP.

The transmission of ventilatory pressures to the intracranial compartment is also influenced by regional lung mechanics. Our EIT-based analysis showed that at high PEEP, the gain in compliance from recruitment outweighed the loss from overdistension, both at the pixel level and across the lung. The RI ratio and Vrec% correlated with the magnitude of ICP changes, indicating that lung recruitability influences the cerebral response to PEEP.

Finally, inhomogeneity in gas distribution (GI) rather than localised lung collapse (LI) was the key factor associated with ICP changes. Large-scale regional inhomogeneity likely exerts a greater influence on pleural pressure and, thus, intracranial dynamics. This highlights that not only the absolute lung recruitability, but also how gas is distributed within the lung affects the transmission of PEEP effects to the brain.

## Strengths and limitations

This study is the first to combine the RI ratio manoeuvre with EIT to explore the link between lung recruitability and ICP. We found a significant association between lung volume recruitment, inhomogeneity of gas distribution, and PEEP impact on ICP. However, several limitations exist: 1) this was a single-centre observational study with a small sample size, intended as a proof-of-concept for future research; 2) we focused on lung mechanics and ICP dynamics, but a more comprehensive haemodynamic and cerebral blood flow analysis is needed; 3) the study included a heterogeneous patient group from a respiratory function perspective, with only three of ten potentially classifiable as ARDS. While this reflects real-world variability, results may not generalise to ARDS populations; 4) the consistent PEEP–ICP effects may be due to all patients being in the early phase of ABI, when cerebral autoregulation is often impaired. Future research should explore how lung recruitability and ICP interactions evolve over time in the Neuro-ICU; 5) neither EIT nor the RI ratio manoeuvre assesses absolute lung inflation or recruitability beyond the tested PEEP range; 6) different ABI diagnoses may affect cerebral blood flow regulation, requiring validation in a larger cohort; 7) variability in ICP devices may introduce minor discrepancies, but focusing on relative changes mitigates this concern.

## Conclusions

This proof-of-concept study suggests that PEEP can adversely affect cerebral arterial inflow despite promoting alveolar recruitment and oxygenation. This was particularly true in patients with higher pulmonary recruitability and greater inhomogeneity in gas distribution. This may result in a paradoxical reduction in ICP, which should be interpreted as a consequence of diminished cerebral arterial blood flow when pressure autoregulation is impaired.

These results challenge existing assumptions and provide novel insights into the intricate interplay between lung mechanics, systemic haemodynamics, and cerebral physiology. Our findings also offer a cautionary note regarding common clinical practice, where a reduction in ICP following changes in ventilator settings—particularly an increase in PEEP—is often regarded as a beneficial outcome of lung recruitment and improved oxygenation.

This study offers novel elements for future studies investigating lung–heart–brain interaction. It suggests that ventilator settings should be individually tailored, considering the complex interaction among the central nervous, respiratory, and cardiovascular systems.

## Supplementary Information

Supplementary file 1.

Supplementary file 2.

## Data Availability

Data are available from the corresponding author upon reasonable request.

## Abbreviations

ABI: Acute brain injury
ARDS: Acute respiratory distress syndrome
AOP: Airway opening pressure
ABP: Arterial blood pressure
CPP: Cerebral perfusion pressure
CVP: Central vein pressure
E_CW_: Elastance of the chest wall
EIT: Electrical impedance tomography
E_L_: Elastance of the lung
E_RS_: Elastance of the respiratory system
EtCO_2_: End-tidal carbon dioxide
F_I_O_2_: Fraction of inspiratory oxygen
Neuro-ICU: Neurosurgical intensive care unit
GI: Global inhomogeneity index
ICP: Intracranial pressure
IQR: Interquartile range
LI: Local inhomogeneity index
MAP: Mean arterial pressure
P2: The second peak
PaCO_2_: Arterial pressure of carbon dioxide
PaO_2_: Pressure of arterial oxygen
PBW: Predicted body weight
PEEP: Positive end-expiratory pressure
Ppl: Transpulmonary pressure
PRx: Pressure reactivity index
RI: Recruitment-to-inflation
SaO_2_: Arterial saturation of oxygen
SD: Standard deviation
Vrec: Recruited volume
Vinfl: Inflated volume
ΔEELV: Delta end-expiratory lung volume
ΔZ: Delta impedance values
