# Supplementary material for: Intracranial response to positive end-expiratory pressure is influenced by lung recruitability and gas distribution during mechanical ventilation in acute brain injury patients: a proof-of-concept physiological study
Source: Intensive Care Med Exp. 2025 Apr 14;13:43. doi: 10.1186/s40635-025-00750-y (PMC11996739; doi:10.1186/s40635-025-00750-y)
Supplement: Supplementary file 1 [file 40635_2025_750_MOESM1_ESM.docx]

**Intracranial response to positive end-expiratory pressure is influenced by lung recruitability and gas distribution during mechanical ventilation in acute brain injury patients: a proof-of-concept physiological study**

Reka Bencze ^1,2*^, Rafael Kawati ^1,2*^, Anders Hånell ^3^, Anders Lewen ^3^, Per Enblad ^3^, Henrik Engquist ^1,2^, Kristin Jona Bjarnadottir ^1,2^, Odin Joensen ^1,2^, Annelie Barrueta Tenhunen ^1,2^, Filip Freden ^1,2^, Laurent Brochard ^4^, Gaetano Perchiazzi ^1,2^, Mariangela Pellegrini ^1,2^

*: shared first authorship

**Affiliations:**

1. Anesthesia, Operation and Intensive Care Medicine, Uppsala University Hospital, Uppsala, Sweden; 2. Hedenstierna Laboratory, Department of Surgical Sciences, Uppsala University, Uppsala, Sweden;

3. Department of Medical Sciences, Section of Neurosurgery, Uppsala University, Uppsala, Sweden.

4. Keenan Centre for Biomedical Research, Critical Care Department, St. Michael’s Hospital, Unity Health Toronto, Toronto, Canada.

**Corresponding author:**

Mariangela Pellegrini, MD, PhD.

Hedenstierna Laboratory, Akademiska sjukhuset ing 40 3 tr, 75185 Uppsala, Sweden. E-mail address: [mariangela.pellegrini@uu.se](mailto:mariangela.pellegrini@uu.se)

**METHODS**

The study was approved by the Swedish National Ethical Review Authority (2020-07227) and conducted following the Helsinki Declaration and its subsequent revisions. This was a preliminary analysis of a more extended study whose protocol was registered a priori (Clinical Trials ID: NCT05363085). The study included mechanically ventilated patients within 72 hours of initiating invasive mechanical ventilation in an early phase after the onset of ABI and receiving PEEP but independently from the severity of their lung condition. Patients were admitted to the neurosurgical intensive care unit (Neuro-ICU) of Uppsala University Hospital, Sweden. As the patients were unconscious at the time of inclusion, informed consent was obtained from the patient’s next of kin. Withdrawal of consent by patients or their legal representatives was possible at any time during the study and until study publication. The study description was sent to the patient's home address, and inclusion was documented in the clinical record, leaving the possibility for future consent withdrawal for patients recovering their cognitive function after the acute illness. Strengthening the Reporting of Observational Studies in Epidemiology (STROBE) guidelines for observational cohort studies were followed to clearly and comprehensively present findings.

*Inclusion and exclusion criteria*

Patients were prospectively screened for eligibility according to inclusion criteria. Inclusion criteria were defined as follows: 1) age higher than 18; 2) ABI within 72 hours from inclusion, i.e., subarachnoid haemorrhage; subdural haemorrhage; intracranial haemorrhage; traumatic brain injury; 3) less than 72 hours since initiation of mechanical ventilation and insertion of invasive intracerebral pressure monitoring device; 3) mechanical ventilation expected to last for longer than 72 hours; 4) ongoing ICP measurement. Exclusion criteria were: 1) chest tube or open chest trauma; 2) absolute contraindication to the insertion of nasogastric catheters: e.g., oesophagus rupture and oesophageal bleeding; 3) contraindication for the use of EIT: pacemaker and implantable cardioverter defibrillator, pregnancy, thoracic skin lesion, and burns on the belt area; 4) hemicraniectomy. A relative contraindication was skull base fracture; in this case, the patient was eligible only if the oesophageal catheters could be inserted orally. In the case of late hemicraniectomy, the patient dropped out of the study.

At inclusion, a nasogastric catheter for oesophagus pressure monitoring (14Fr, Nutrivent, Sidam) and the thinnest catheter for measurement of the electrical activity of the diaphragm (8Fr/125cm, Edi, Getinge) were placed. At the moment of data acquisition, the oesophageal catheter and a patient spirometry catheter (D-lite+, GE Healthcare) with only 9.5 ml instrumental dead space not significantly interfering with patient CO_2_ clearance were connected to the Pulmovista 500 pressure transducer (Pressure Pod, Dräger). Based on clinical routine, all patients had a central line catheter for drug infusion and central vein pressure (CVP) monitoring and an arterial catheter for invasive systemic blood pressure monitoring. Following clinical practice for intubated patients, side-stream capnography was recorded, and end-tidal carbon dioxide (EtCO_2_) was continuously monitored. All respiratory, hemodynamic, and neuro-monitoring variables, including EIT imaging, were simultaneously acquired and synchronised offline for data analysis using MatLab, R2023b (The Mathworks, Natick, MA, USA). Demographics, epidemiologic, and clinical data were obtained from electronic medical records.

*Patient management during data collection*

For data acquisition, all patients were placed in a semi-recumbent 30° head-elevated position and continuously sedated according to local standard practice. Deep sedation (corresponding to Richmond Agitation Sedation Scale -4 or lower) and complete neuromuscular blockade (induced by repeated rocuronium boluses 0.5mg/kg and confirmed by no trigger on the ventilator and the absence of organised electrical activity of the diaphragm) were established before data acquisition. The intraventricular drain catheter was closed throughout the protocol for ICP recording. An EIT belt was placed to collect chest impedance changes related to lung ventilation (Pulmovista 500, Dräger, Germany). The EIT belt was in place only during data acquisition and then removed, not interfering with clinical procedures. We strictly aligned with neuroprotection care bundle principles and always prioritised patient safety and best care. The protocol was performed by an experienced research team with extensive experience in neurointensive care who were trained to ensure minimal patient disturbance. Data collection was planned during periods of clinical stability, with interventions synchronised with standard care procedures to avoid unnecessary handling. To minimise interference with clinical activities, data were collected during the afternoon. The protocol was well-known to the entire treating team. Before data acquisition, the attending intensivist, neurosurgeon and nurse were consulted to detect possible instabilities and contraindications to data collection. Data were not collected in cases of severe respiratory (fraction of inspiratory oxygen - F_I_O_2_ - higher than 75% to reach a partial pressure of arterial oxygen - PaO_2_ - of 80 mm Hg and respiratory rate higher than 35 breath/min), hemodynamic (heart rate higher than 150/min and mean arterial pressure lower than 60 mm Hg during continuous vasopressor support), or neurologic (sustained mean ICP higher than 15 mm Hg or strict control of partial pressure of carbon dioxide - PaCO_2_ - range for neurosurgical reasons) instability.

*Protocol and data collection*

The ventilation mode was first changed to volume-controlled ventilation with a tidal volume of 6-8 ml/kg predicted body weight. The respiratory rate was adjusted to keep EtCO_2_ constant and maintain the clinical target of PaCO_2_ unchanged. F_I_O_2_ was set to stay PaO_2_ higher than 90 mm Hg. PEEP was kept as previously clinically set. Before data acquisition, oesophageal catheter positioning, adequate sedation level and muscle relaxation were checked. Data recording was initiated, and a standardised lung recruitment manoeuvre was performed, followed by a decreasing PEEP titration. Lung recruitment consisted of a gradual increase in PEEP, in steps of 2 cm H_2_O, to a maximum PEEP of 20 cm H_2_O or a maximum peak airway pressure of 40 cm H_2_O, depending on which one was reached first. Baseline PEEP was set based on best static compliance. After PEEP titration, an inspiratory-hold and an expiratory-hold manoeuvre were performed. A low-flow (5  L/min) inflation starting from PEEP 0 cm H_2_O and reaching the patient’s set tidal volume was performed to identify airway opening pressure (AOP), as previously described [1]. After baseline ventilation and reduction of respiratory rate to 10 breaths per minute to exclude the onset of intrinsic PEEP, patients were exposed to a single-breath de-recruitment manoeuvre from high to low PEEP, with a delta of 10 cm H_2_O, to calculate the RI ratio as previously described [2, 3]. Absolute PEEP values (e.g., 15/5 cmH₂O or 12/2 cmH₂O) were tailored to individual patient tolerance, and low PEEP was always set above AOP. Each PEEP step was set for at least three minutes, and plateau pressure was measured at low PEEP at the end of the manoeuvre. The RI ratio was spirometrically computed, and EIT images were acquired for a subsequent offline analysis of the recruited and inflated volumes. EIT images and other variables were selected during steady-state conditions for each PEEP level.

*Respiratory variables*

The end-expiratory transpulmonary pressure (Ppl) was directly calculated by subtracting Pes from PEEP [4]. This estimation of Ppl greatly reflects the dependent lung regions and is, as such, more representative of lung collapse [5]. Given its importance in promoting the transmission of static respiratory pressures to the intrathoracic and intracranial cavities [6, 7], chest wall elastance and its ratio to respiratory system elastance (E_CW_/E_RS_), as well as lung elastance (E_L_) where E_RS_ = E_CW_+E_L_, were calculated. Given its clinical impact in mechanically ventilated patients with ABI [8], mechanical power was calculated as in Gattinoni et al. [9].

*Neuromonitoring variables*

Cerebral perfusion pressure (CPP) was calculated by subtracting ICP from mean arterial pressure (MAP). The pressure reactivity index (PRx), an indicator for cerebral autoregulation, was calculated as previously described [10]: a moving correlation coefficient between MAP and ICP averaged over 10 s, using a 5-minute moving time window. Therefore, the values collected during a time interval of 1 hour before protocol start were averaged. A PRx value higher than 0.2 reflects poor cerebral autoregulation. The ICP pulse waveform analysis in the time domain was conducted to identify the second peak (P2), also called the tidal wave of the ICP curve, which is a proxy for intracranial compliance [11].

*Hemodynamics and other variables*

MAP was calculated as the sum between 1/3(systolic blood pressure) and 2/3(diastolic blood pressure). Arterial and central venous blood samples were collected three times during the protocol: 1) at least five minutes after the lung recruitment manoeuvre, 2) at high PEEP, and 3) at low PEEP during RI manoeuvre. Based on blood gas analysis, the ventilatory ratio, estimating the dead space fraction, was calculated as: (minute ventilation [ml/min] × PaCO_2_ [mm Hg]) / (PBW × 100 [ml/min] × 37.5 [mm Hg]) [12]. The shunt fraction was estimated based on the venous admixture determination, considering the central venous oxygen saturation as an acceptable surrogate for mixed venous oxygen saturation and assuming the respiratory ratio equal to 0.8 [13].

Figure 1 provides a schematic representation of the protocol.

**Data analysis**

All ventilation-related data, including lung mechanics recorded from the ventilator (SERVO Tracker, Getinge, Sweden) and the EIT equipment (Pulmovista 500 and PressurePod, Dräger, Germany) as well as the neuromonitoring, the capnography curve and the hemodynamic data collected from the bedside monitors (IntelliVue MX, Philips) were off-line synchronised based on the simultaneous acquisition of the airway pressure curve on all devices. After synchronisation, the final sample rate was 50 Hz. The whole data analysis was performed using custom scripts developed by one of the authors (MP) using the MatLab platform (MATLAB R2023b, MathWorks, Natick, MA, USA).

***EIT analysis.*** *EIT and volume maps derived during the RI ratio manoeuvre.* The EIT baseline was set to correspond to the dynamic image with the lowest global impedance during expiratory hold at low PEEP. After that, dynamic EIT images (32x32 matrix) corresponding to 1) inspiratory hold PEEP low and 2) expiratory hold PEEP high were selected. For both selected EIT images, pixels characterised by delta impedance values (ΔZ) below 20% of the highest ΔZ in the same EIT image were considered not ventilated [14, 15]. Based on the set tidal volume, impedance changes were translated into corresponding millilitres of gas to obtain the regional (pixel-based) distribution of ventilation [16]. Based on these EIT-derived images and the physiological rationale behind the RI ratio manoeuvre, the following three spirometric volume and volume maps, the latter providing pixel-wise information, were computed (see Figures 1 and 2):

1. **Delta end-expiratory lung volume (****ΔEELV)**: the global change in lung volume corresponding to a delta PEEP of 10 cm H_2_O, between PEEP high and PEEP low and calculated as follows:

**ΔEELV** = (EIT image during expiratory hold PEEP high) – (EIT image during expiratory hold PEEP low)

Where (EIT derived image during expiratory hold PEEP low) corresponds to the EIT baseline.

1. **Inflated volume (****Vinfl)**: the portion of ΔEELV inflating the already opened lung when going from PEEP low to PEEP high and calculated as:

**Vinfl** = [(EIT image during inspiratory hold PEEP low - EIT image during expiratory hold PEEP low)/ (plateau pressure – PEEP low)] x (PEEP high – PEEP low)

Where [(EIT image during inspiratory hold PEEP low - EIT image during expiratory hold PEEP low)/ (plateau pressure – PEEP low)] is the compliance of the lung open at PEEP low.

1. **The recruited** **volume (Vrec)**: the portion of ΔEELV recruiting new portions of lung parenchyma when going from PEEP low to PEEP high and calculated as:

**Vrec** = ΔEELV- Vinfl

This way, information about the distribution of inflated and recruited volumes was calculated globally and regionally (at a pixel level) for each included patient. The Vrec was then expressed as a percentage (Vrec%) of the corresponding ΔEELV.

***EIT analysis.*** *Compliance gain between the two tested PEEP.* To further investigate lung recruitability and confirm lung recruitment and inflation at the two tested PEEP, regional maps of compliance at both low and high PEEPs have been computed based on the tidal impedance changes between plateau pressure and the corresponding PEEP. The estimated tidal volume per pixel was then divided by the measured driving pressure to obtain the compliance maps (Figure E1). Subsequently, the compliance map at high PEEP was subtracted from the compliance map at low PEEP to obtain the distribution of differential compliance and, as such, identify pixels of compliance gain from pixels of compliance loss. The number of pixels with compliance gain was then expressed as a percentage of all pixels covering the lung area. Moreover, the mean compliance gain (or loss) was expressed in [ml/cm H_2_O per pixel]. The pixel compliance gain (or loss) summation expressed as [ml/cm H_2_O] was subsequently calculated.

***EIT analysis.*** *EIT-based inhomogeneity index.* To investigate the coupling between regional inhomogeneity of lung ventilation and neuromonitoring variables, two EIT-based indices were calculated for the ΔEELV delta volume maps: the global inhomogeneity index (GI) and the local inhomogeneity index (LI) [17]. GI shows the variation of tidal volume distribution in the whole lung. GI is calculated as the summation of the absolute difference between the median value of the estimated gas volume for each analysed map and the estimated gas volume for every pixel. LI calculates the local differences among neighbour pixels. GI and LI were normalised by dividing them by the total gas content in the analysed volume map to make both indices comparable among patients.

GI and LI can be summarised with the following equations as in Zhao et al. [17]:

$$GI =\sum_{x,y \in lung} \left| {Dgas}_{xy}-Median ({Dgas}_{lung}) \right| \div\sum_{x,y \in lung} {Dgas}_{xy}$$

$$LI =\sum_{x,y \in lung} \left( \frac{1}{m-1}\times\sum_{i,j \in\left\lfloor-1,1 \right\rfloor\cap x+1, y+i \in lung} \left| {Dgas}_{xy}-{Dgas}_{x+i, y+j} \right| \right) \div\sum_{x,y \in lung} {Dgas}_{xy}$$

Where *x* and *y* are the coordinates of each pixel; Dgas_xy_ is the value of the differential impedance for the single pixel; Median (Dgas_lung_)is the median value of impedance in the lung area; m-1 is the number of neighbour pixels equal to eight; Dgas_x+i,y+j_ is the differential impedance for the neighbouring pixels of Dgas_xy_. These two indices report different information about gas distribution. GI is an index of global lung inhomogeneity, while LI quantifies local inhomogeneity among neighbour pixels.

**Neuromonitoring.** A catheter for continuous invasive ICP monitoring (intraparenchymal or external ventricular drain) has been inserted based on patients’ needs and according to local clinical practice. In the current study, to investigate the association between ventilatory variables (i.e., percentage of recruitable lung and indices of regional inhomogeneity) and neuromonitoring variables, we referred to changes of ICP and CPP (ΔICP, ΔCPP) going from low PEEP to high PEEP levels, positive changes indicating a worse ICP dynamics at low PEEP compared to high PEEP.

**Statistical analysis**

An a priori sample size calculation was not feasible as no studies investigated the association between EIT-derived indices of lung recruitability and intracranial pressure in brain-injured patients. Our sample size was comparable to previous physiologic studies regarding PEEP changes in ABI patients [18]. The primary outcome was to test the association between recruited volume and ΔICP and the association between inhomogeneity (GI, LI) in gas distribution in the ΔEELV and ΔICP. Data were expressed as median and interquartile range (interquartile range, IQR) or mean (± standard deviation, SD). The correlation between different variables was assessed using Spearman’s rank correlation coefficient with a 95% confidence interval. F-test statistic (α=0.05) was used for linear regression analysis. Wilcoxon matched-pairs signed-rank test was used to test statistically significant changes in respiratory and neuromonitoring variables between the two tested PEEP levels. In case of missing values in the data range, the whole pair was excluded from the analysis. Friedman's test, followed by Bonferroni’s correction, was used to test statistical differences for neuromonitoring parameters among baseline ventilation, high PEEP and low PEEP. Statistical analysis was performed using MatLab (MATLAB R2023b, MathWorks, MA, USA) and GraphPad (GraphPad Prism v10, California, USA).

**REFERENCES**

1. Chen L, Del Sorbo L, Grieco DL, et al (2018) Airway Closure in Acute Respiratory Distress Syndrome: An Underestimated and Misinterpreted Phenomenon. Am J Respir Crit Care Med 197:132–136. https://doi.org/10.1164/rccm.201702-0388LE

2. Chen L, Del Sorbo L, Grieco DL, et al (2020) Potential for Lung Recruitment Estimated by the Recruitment-to-Inflation Ratio in Acute Respiratory Distress Syndrome. A Clinical Trial. Am J Respir Crit Care Med 201:178–187. https://doi.org/10.1164/rccm.201902-0334OC

3. Dellamonica J, Lerolle N, Sargentini C, et al (2011) PEEP-induced changes in lung volume in acute respiratory distress syndrome. Two methods to estimate alveolar recruitment. Intensive Care Med 37:1595–1604. https://doi.org/10.1007/s00134-011-2333-y

4. Gattinoni L, Chiumello D, Carlesso E, Valenza F (2004) Bench-to-bedside review: chest wall elastance in acute lung injury/acute respiratory distress syndrome patients. Crit Care 8:350–355. https://doi.org/10.1186/cc2854

5. Yoshida T, Amato MBP, Grieco DL, et al (2018) Esophageal Manometry and Regional Transpulmonary Pressure in Lung Injury. Am J Respir Crit Care Med 197:1018–1026. https://doi.org/10.1164/rccm.201709-1806OC

6. Chen H, Zhou J, Lin Y-Q, et al (2018) Intracranial pressure responsiveness to positive end-expiratory pressure in different respiratory mechanics: a preliminary experimental study in pigs. BMC Neurol 18:183. https://doi.org/10.1186/s12883-018-1191-4

7. Chen H, Chen K, Xu J-Q, et al (2018) Intracranial pressure responsiveness to positive end-expiratory pressure is influenced by chest wall elastance: a physiological study in patients with aneurysmal subarachnoid hemorrhage. BMC Neurol 18:1–8. https://doi.org/10.1186/s12883-018-1132-2

8. Wahlster S, Sharma M, Taran S, et al (2023) Utilization of mechanical power and associations with clinical outcomes in brain injured patients: a secondary analysis of the extubation strategies in neuro-intensive care unit patients and associations with outcome (ENIO) trial. Crit Care 27:1–16. https://doi.org/10.1186/s13054-023-04410-z

9. Gattinoni L, Tonetti T, Cressoni M, et al (2016) Ventilator-related causes of lung injury: the mechanical power. Intensive Care Med 42:1567–1575. https://doi.org/10.1007/s00134-016-4505-2

10. Czosnyka M, Smielewski P, Kirkpatrick P, et al (1997) Continuous Assessment of the Cerebral Vasomotor Reactivity in Head Injury. Neurosurgery 41:11

11. Cucciolini G, Motroni V, Czosnyka M (2023) Intracranial pressure for clinicians: it is not just a number. J Anesth Analg Crit Care 3:31. https://doi.org/10.1186/s44158-023-00115-5

12. Sinha P, Calfee CS, Beitler JR, et al (2019) Physiologic Analysis and Clinical Performance of the Ventilatory Ratio in Acute Respiratory Distress Syndrome. Am J Respir Crit Care Med 199:333–341. https://doi.org/10.1164/rccm.201804-0692OC

13. Tahvanainen J, Meretoja O, Nikki P (1982) Can central venous blood replace mixed venous blood samples? Crit Care Med 10:758–761. https://doi.org/10.1097/00003246-198211000-00012

14. Pulletz S, van Genderingen HR, Schmitz G, et al (2006) Comparison of different methods to define regions of interest for evaluation of regional lung ventilation by EIT. Physiol Meas 27:S115-127. https://doi.org/10.1088/0967-3334/27/5/S10

15. Becher T, Vogt B, Kott M, et al (2016) Functional Regions of Interest in Electrical Impedance Tomography: A Secondary Analysis of Two Clinical Studies. PLoS One 11:e0152267. https://doi.org/10.1371/journal.pone.0152267

16. Hinz J, Neumann P, Dudykevych T, et al (2003) Regional Ventilation by Electrical Impedance Tomography: A Comparison With Ventilation Scintigraphy in Pigs. Chest 124:314–322. https://doi.org/10.1378/chest.124.1.314

17. Zhao Z, Möller K, Steinmann D, Guttmann J (2009) Global and local inhomogeneity indices of lung ventilation based on electrical impedance tomography. In: Vander Sloten J, Verdonck P, Nyssen M, Haueisen J (eds) 4th European Conference of the International Federation for Medical and Biological Engineering. Springer, Berlin, Heidelberg, pp 256–259

18. Robba C, Ball L, Nogas S, et al (2021) Effects of Positive End-Expiratory Pressure on Lung Recruitment, Respiratory Mechanics, and Intracranial Pressure in Mechanically Ventilated Brain-Injured Patients. Front Physiol 12:711273. https://doi.org/10.3389/fphys.2021.711273
