# Supplementary material for: Intracranial response to positive end-expiratory pressure is influenced by lung recruitability and gas distribution during mechanical ventilation in acute brain injury patients: a proof-of-concept physiological study
Source: Intensive Care Med Exp. 2025 Apr 14;13:43. doi: 10.1186/s40635-025-00750-y (PMC11996739; doi:10.1186/s40635-025-00750-y)
Supplement: Supplementary file 2 [file 40635_2025_750_MOESM2_ESM.pdf]

Intracranial response to positive end-expiratory pressure is influenced by lung recruitability and gas distribution during mechanical ventilation in acute brain injury patients: a proof-of-concept physiological study

Reka Bencze, Rafael Kawati, Anders Hånell, Anders Lewen, Per Enblad, Henrik Engquist, Kristin Jona Bjarnadottir, Odin Joensen, Annelie Barrueta Tenhunen, Filip Freden, Laurent Brochard, Gaetano Perchiazzi, Mariangela Pellegrini

Supplementary material

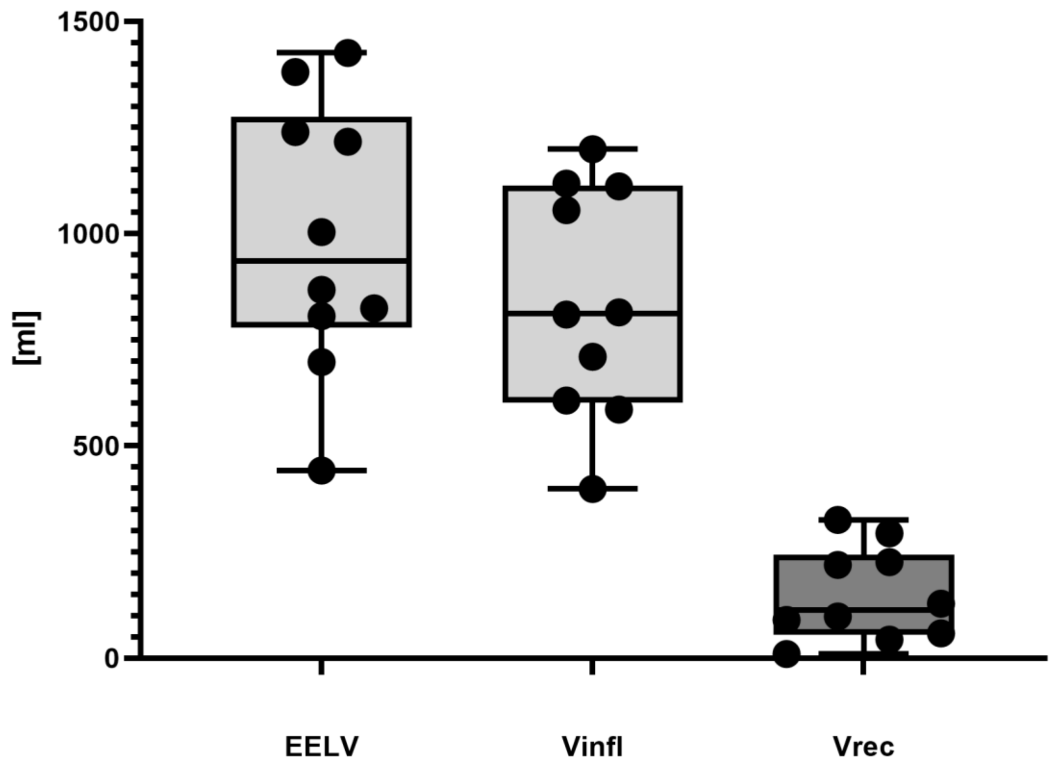

Figure E1. Spirometric delta end-expiratory lung volume (**Δ**EELV), inflated volume (Vinfl) and recruited volume (Vrec). ΔEELV, Vinfl, and Vrec calculated the recruitment-to-inflation ratio based on the single-breath de-recruitment manoeuvre from high to low PEEP.

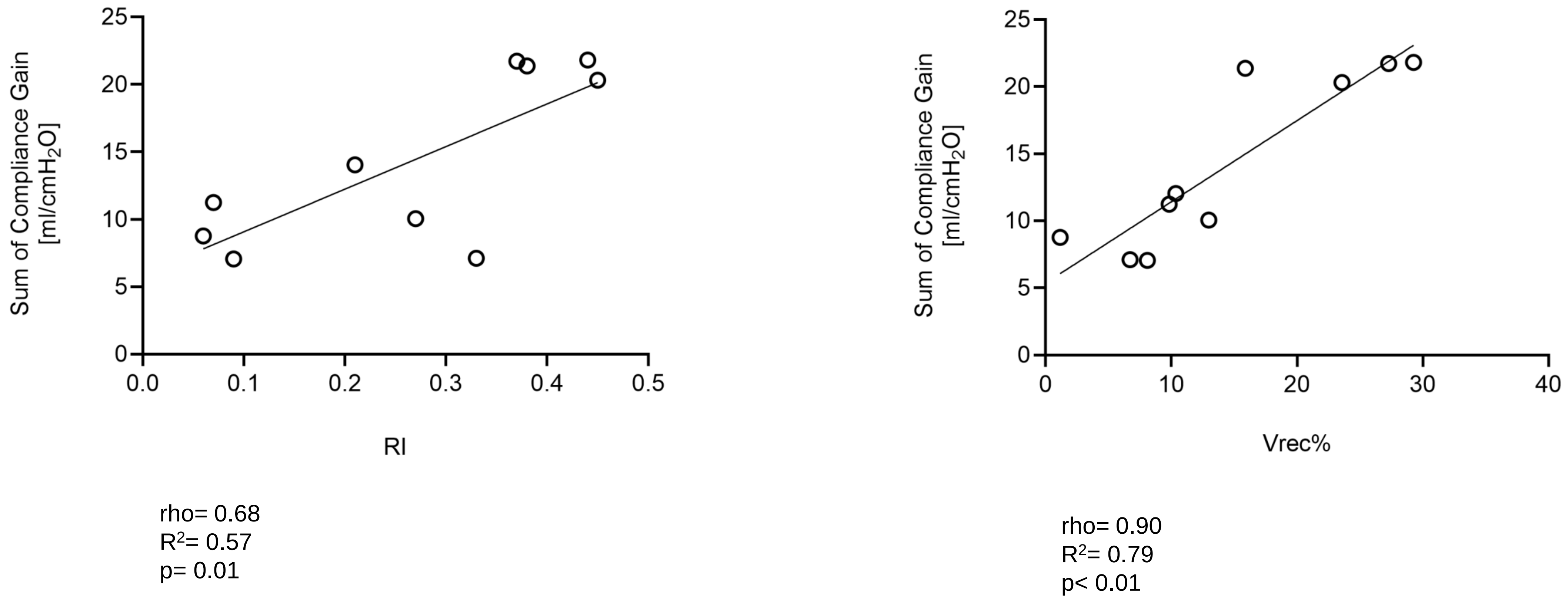

Figure E2. Linear regression and Spearman's correlation investigating the relationship between the sum of Compliance Gain [ml/cmH_2O] and a) RI ratio or b) Vrec%. A sensitivity analysis to investigate the relationship between the indicators of recruitment reported in the study (spirometric RI ratio and Vrec%) and the regional gain in compliance. RI ratio: recruitment-to-inflation ratio; Vrec%: recruited volume expressed as a percentage of the corresponding ΔEELV.

Patient number	FIO_2	PaO_2 Baseline	PaO_2 HIGH PEEP	PaO_2 LOW PEEP	PaO_2/FIO_2 Baseline	PaO_2/FIO_2 HIGH PEEP	PaO_2/FIO_2 LOW PEEP	ΔPaO2/FIO2 (LOW PEEP - HIGH PEEP)	RI
1	0.30	113	113		375	378			0.07
2	0.30	139	139		463	463			0.38
3	0.30	129	124		430	413			0.33
4	0.30	100	107	89	333	355	298	-58	0.27
5	0.55	98	124	105	179	225	191	-35	0.45
6	0.50	101	100	113	203	200	225	25	0.06
7	0.35	109	95	91	311	272	259	-13	0.21
8	0.30	105	110	95	350	365	315	-50	0.44
9	0.60	99	100	79	165	167	131	-35	0.37
10	0.30	142	136	145	473	453	483	30	0.09

Table E1. Individual FIO_2, PaO_2, PaO_2/FIO_2 and spirometrical RI ratio values. Three out of ten patients had missing values for PaO_2 at low PEEP. FIO_2: fraction of inspiratory oxygen; PaO_2: arterial partial pressure of oxygen; RI: recruitment-to-inflation ratio; rho = Spearman correlation coefficient; R^2: coefficient of determination; p = p-value or probability value.

Neuromonitoring variables	Baseline median	Baseline IQR	PEEP high median	PEEP high IQR	PEEP low median	PEEP low IQR	Friedman's test p-value	Baseline vs High PEEP p-value	Baseline vs Low PEEP p-value	High PEEP vs Low PEEP p-value
CPP [mmHg]	79	[70 - 84]	71	[67 - 83]	75	[70 - 84]	0.03 *	0.29	1	0.03 *
ICP [mmHg]	7	[4 - 10]	9	[5 - 13]	12	[8 - 16]	< 0.01 *	0.53	< 0.01 *	0.02 *
P2 [mmHg]	12	[10 -14]	12	[10 -15]	15	[12 - 21]	< 0.01 *	0.79	< 0.01 *	0.04 *

Table E2. Friedman's test followed by Bonferroni's correction for comparisons among neuromonitoring variables. 1) baseline ventilation, 2) PEEP high, and 3) PEEP low. Data reported as median [interquartile range]. Abbreviations: CPP: cerebral perfusion pressure; ICP: intracranial pressure; P2, ICP: second peak of intracranial pressure; IQR: interquartile range. *: to mark significant differences.

RI associated variables	mean	[± STD]
Regional (pixelwise)		
ΔEELV [ml]	3.7	[± 1.3]
Inflating Volume [ml]	3.1	[± 1]
Recruiting Volume [ml]	0.7	[± 0.5]
Recruiting Volume [% of ΔEELV]	16	[± 10]

Table E3. Inflated and Recruited volumes. Delta end-expiratory lung volume (ΔEELV), inflated volume (Vinfl) and recruited volume (Vrec) were computed at a pixel level. Data reported as mean [± STD]. Abbreviations: RI: recruitment inflation ratio; ΔEELV: delta of end-expiratory lung volume between the two tested PEEP levels; GI: global inhomogeneity index; LI: local inhomogeneity index; STD: standard deviation.
